# Autonomic dysregulation, cognition and fatigue in people with depression and in active and healthy controls: observational cohort study

**DOI:** 10.1192/bjo.2023.68

**Published:** 2023-06-14

**Authors:** Tiago Costa, Abigail Taylor, Francesca Black, Sean Hill, R. Hamish McAllister-Williams, Peter Gallagher, Stuart Watson

**Affiliations:** Translational and Clinical Research Institute, Faculty of Medical Sciences, Newcastle University, Newcastle upon Tyne, UK; Northern Centre for Mood Disorders, Newcastle University, Newcastle upon Tyne, UK; and Cumbria, Northumberland, Tyne and Wear NHS Foundation Trust, Newcastle upon Tyne, UK; Translational and Clinical Research Institute, Faculty of Medical Sciences, Newcastle University, Newcastle upon Tyne, UK; Translational and Clinical Research Institute, Faculty of Medical Sciences, Newcastle University, Newcastle upon Tyne, UK; and Northern Centre for Mood Disorders, Newcastle University, Newcastle upon Tyne, UK; Translational and Clinical Research Institute, Faculty of Medical Sciences, Newcastle University, Newcastle upon Tyne, UK; Northern Centre for Mood Disorders, Newcastle University, Newcastle upon Tyne, UK; and Cumbria, Northumberland, Tyne and Wear NHS Foundation Trust

**Keywords:** Depressive disorders, autonomic, fatigue, cognition, medication

## Abstract

**Background:**

Autonomic nervous system (ANS) dysregulation might be relevant to the pathophysiology of fatigue and cognitive impairment in depression and perhaps should be considered when making prescribing decisions.

**Aims:**

To determine the relationship of self-reported ANS symptoms with fatigue, cognition and prescribed medication in people with a diagnosis of depression, in comparators without depression but with other mental health, neurodevelopmental or neurodegenerative disorders (active controls) and in healthy controls.

**Method:**

Cross-sectional analysis of an opportunistic sample from England. Self-reported data were collected on demographics, diagnosis, medication, ANS symptoms (Composite Autonomic Symptom Scale-31, COMPASS-31) and fatigue (Visual Analogue Scale for Fatigue, VAS-F). A subsample completed cognitive tests (THINC-it), including the subjective Perceived Deficits Questionnaire five-item version (PDQ-5). Spearman's correlation and mediation models were used to explore the relationship between COMPASS-31, VAS-F and PDQ-5 scores.

**Results:**

Data were obtained for 3345 participants, 22% with depression. The depression group had significantly (*P* < 0.01) more severe autonomic dysregulation as measured by COMPASS-31 scores (median 30) than active (median 23) and healthy controls (median 10). The depression group had significantly higher symptom severity (*P* < 0.01) than both control groups on the VAS-F and PDQ-5. Overall, there was a significantly positive correlation (*P* < 0.01) between COMPASS-31, VAS-F scores (Spearman's rho *r*_s_ = 0.44) and PDQ-5 scores (*r*_s_ = 0.56). COMPASS-31 scores mediated greater symptom severity on the VAS-F and PDQ-5 for those with depression. COMPASS-31 scores remained significantly different between the depression group and both control groups independently of medication.

**Conclusions:**

People with a diagnosis of depression report worse fatigue and cognition than active and healthy comparators; this appears to be mediated by ANS dysregulation.

The autonomic nervous system (ANS) unconsciously regulates bodily functions, including the cardiovascular, respiratory, endocrine and digestive systems. It relies on a dynamic interplay between the sympathetic and the parasympathetic nervous systems:^1^ the sympathetic nervous system mostly handles the ‘fight-or-flight’ response to stressors and the parasympathetic the ‘rest and digest’ functions. There are disorders – such as postural orthostatic tachycardia syndrome (POTS) – in which ANS dysregulation is a core feature.^2^ There is also increasing support for the notion that more subtle ANS dysregulation is important in fibromyalgia,^3^ chronic fatigue syndrome,^4^ Sjögren's syndrome^5^ and Lewy body disorders.^6^ Objective measures of ANS dysregulation – such as reduced heart rate variability (HRV) – predict cardiovascular morbidity and mortality^7^ and have been described in studies in depression and other psychiatric disorders.^8^ Other objective biomarkers of ANS function used in research include baroreflex sensitivity, skin conductivity and brain functional imaging.^9^ Depression is also associated with cognitive impairment.^10^ Together with the demonstrated correlations between the severity of ANS dysregulation and the degree of cognitive impairment^11^ as seen, for example, in POTS,^2^ and the association of ANS dysregulation with fatigue severity in chronic fatigue syndrome,^12^ a number of questions arise regarding the relationship between ANS dysregulation and depression. The first question is whether ANS dysregulation might be relevant to the pathophysiology of low mood,^8^ fatigue^13^ and cognitive impairment^14^ in those with a diagnosis of depressive disorder. Relevant evidence for the scientific merit of this question comes from other conditions where ANS dysregulation is relatively better characterised. For example, ANS dysregulation significantly correlates with comorbid depression and fatigue levels in Sjögren's syndrome^5^ and with cognitive impairment in Lewy body pathologies.^15^ Further questions relate to the role of psychotropic medication in potentially exacerbating ANS dysregulation and the associated symptoms,^16^ the fundamental issue being that of clarifying to what degree medication contributes with cholinergic burden and therefore muddles the putative association between mood disorders and ANS dysregulation. Answering these questions has relevance to understanding the pathophysiology of mood and cognitive disorders, as well as to the development of targeted therapeutic interventions.

The CAP-MEM (Cause and Prevalence of Memory Problems in People with Mental Health Disorders) study was designed to recruit an observational cohort, with the objective of determining whether symptoms of ANS dysfunction are more prevalent in those with diagnosed mental health conditions and assessing whether symptoms of ANS dysfunction are associated with markers of disease severity, fatigue, cognitive dysfunction and medication use. We have examined the relationship of self-reported ANS symptoms with fatigue, cognitive performance and prescribed medication in people with depression, in comparators with other mental health, neurodevelopmental or neurodegenerative disorders (active controls) and in healthy controls.

## Method

### Participants

The CAP-MEM study had no specific diagnosis or symptoms defined as inclusion criteria. The only defined inclusion criteria were age over 16 years and fluency in the English language. Participants could have any diagnosis or no diagnosis at all. As the opportunity sample was recruited via self-referral from secondary care National Health Service (NHS) mental health trusts and via the National Institute for Health and Care Research (NIHR) Clinical Research Network (CRN) teams across England, a high proportion of participants with mental health diagnoses was expected. Healthy matched controls were recruited via databases of volunteers at Newcastle University and by advertisement. Written informed consent was obtained from all participants. Capacity to provide and communicate informed consent was assumed unless there was a reason to believe that capacity was impaired. In such circumstances capacity was assessed face to face by a member of the research team. Potential participants who demonstrated a lack of capacity were not eligible to participate. The authors assert that all procedures contributing to this work comply with the ethical standards of the relevant national and institutional committees on human experimentation and with the Helsinki Declaration of 1975, as revised in 2008. All procedures involving human subjects/patients were approved by the South-Central Hampshire B Research Ethics Committee (Integrated Research Approval System number 226258). Participants did not receive any financial inducement to participate.

### Study design and instruments

Participants completed questionnaires, using an online form or using pen and paper, on demographics (gender, age and ethnicity), self-reported diagnosis, currently prescribed medication, ANS function and fatigue severity. An opt-in subsample completed cognitive function tests. Participants could complete the questionnaires alone, supported by a friend or family member or supported by a member of their clinical team, the research team or a CRN team. This was not controlled. On the study questionnaire each diagnosis was classified independently – for example, a participant could select depression and bipolar affective disorder separately – and all reported diagnoses were coded separately in the study database. Therefore, participants could have more than one diagnosis coded. The diagnoses listed included organic disorders (dementia, mild cognitive impairment and other neurodegenerative disorders), disorders due to psychoactive substance use (alcohol use disorder, other substance use disorders), psychotic disorders (schizophrenia, other psychotic disorders), mood and anxiety disorders (depression, bipolar disorder, generalised anxiety disorder, panic disorder, obsessive–compulsive disorder and other anxiety disorders), personality disorders (borderline personality disorder, other personality disorders), neurodevelopment disorders (autism spectrum disorder, attention-deficit hyperactivity disorder and other neurodevelopmental disorders) and eating disorders. Participants could select multiple disorders from the list. There was also an option for participants to input ‘other’ diagnoses in a free-text box. Clinical severity was estimated by a member of the research team (supported as necessary by the participants' clinical team and/or healthcare records) using the Clinical Global Impression – Severity scale (CGI),^17^ the only clinician-rated instrument applied. Higher scores on the CGI indicate higher overall clinical severity. Current medication was self-reported. ANS function was assessed using the self-reported Composite Autonomic Symptom Scale-31 (COMPASS-31),^18^ which is comprised of six domains: orthostatic intolerance, and secretomotor, vasomotor, gastrointestinal, bladder and pupillomotor function. Higher scores on the COMPASS-31 indicate greater severity of autonomic symptomatology. Fatigue severity was assessed using the Visual Analogue Scale for Fatigue (VAS-F),^19^ consisting of a 100 mm horizontal line with written descriptions at each end (‘no fatigue’ on the left and ‘unberable fatigue’ on the right): participants are asked to mark on the line the point that they feel represents their level of fatigue. The VAS-F scores ranged from 0 to 100, measured in millimetres. Higher scores on the VAS-F indicate more severe fatigue. In the opt-in subsample, cognitive function was assessed using the THINC-it tool.^20^ This includes the five-item version of the Perceived Deficits Questionnaire for Depression (PDQ-5), a self-rated measure of cognitive function (in relation to attention/concentration, planning/organisation, and retrospective and prospective memory). There are also four traditional cognitive assessments which have been reconfigured, gamified and validated^20^ for computer-based administration using the THINC-it tool: choice reaction time (CRT) as a measure of attention; ‘one-back’ paradigm (*N*-back) as a measure of working memory; Part B of the Trail Making Test (TMT-B) as a measure of executive function; and the Digit Symbol Substitution Test (DSST), which tests various domains, including processing speed, working memory and attention/executive function. Higher scores on the PDQ-5 indicate more severe self-perceived cognitive deficits. For the THINC-it objective tests, average reaction times and total number of items correct are reported.

### Data management and analysis

Initial data preparation and cleaning was performed on anonymised and coded quantitative data. The free-text box for ‘other’ diagnosis was checked manually for relevant information. The diagnoses of post-traumatic stress disorder (PTSD), stroke, epilepsy, multiple sclerosis and Parkinson's disease were coded manually into the study database. For this analysis, participants who reported depression in the study questionnaire were included in the ‘depression’ group, independently of any other comorbid diagnosis coded. For example, a participant who reported ‘depression’ and ‘bipolar affective disorder’ was coded in the ‘depression group’ for this analysis. Participants who did not report depression but reported at least one of the other listed diagnoses, or any of the manually coded diagnoses in the free-text box, were included in the ‘active controls’ group. Participants who did not report any of the originally listed diagnoses or any of the manually coded diagnoses in the free-text box were classed as ‘healthy controls’. No information was collected on whether reported diagnoses were ‘active’ or ‘historical’. Current medication was coded into psychotropic subtype (‘antidepressant’, ‘antipsychotic’, ‘mood stabiliser’, ‘sedatives and anxiolytics’ or ‘other’ psychotropic drugs drugs). A category of ‘anticholinergic medication’ was constructed, containing medications known to have higher anticholinergic burden: the selective serotonin reuptake inhibitor (SSRI) paroxetine, all the tricyclic antidepressants (TCAs), the antipsychotics chlorpromazine, clozapine and olanzapine, and the sedative promethazine. Beta-blockers were also coded manually into a separate category.

Age, COMPASS-31 weighted score, VAS-F score and total score for each of the five neuropsychological tasks from the THINC-it tool were not normally distributed and therefore non-parametric Mann–Whitney and Kruskal–Wallis tests were used for between-group comparisons for continuous variables. An alpha level of 0.05 was used throughout. Effect sizes were calculated using eta-squared for continuous variables and Cramér's *V* for contingency tables. Spearman's correlation was used to investigate the relationship between COMPASS-31, VAS-F and PDQ-5 scores. Hierarchical multiple regressions were used to analyse the effects on objective THINC-it measures (the dependent variables) of COMPASS-31 scores (step 1) and group membership (added at step 2). For the multiple regression analyses group membership was coded using dummy variables, with the depression group being the reference variable. Mediation models were constructed using the PROCESS macro version 4.1 for SPSS for Mac OS,^21^ with diagnoses groups being used as a categorical independent variable,^22^ COMPASS-31 scores as the mediation variable, and VAS-F and PDQ-5 scores as dependent variables. The selection of the dependent variables for the mediation models was informed by the results of the Spearman's correlations. IBM SPSS Statistics version 27 for Mac OS was used for analysis.

## Results

Data were obtained for 3345 participants. A depression diagnosis was self-reported by 740 participants (22%) and all were included in the ‘depression’ group. There were two control groups: 953 (29%) active controls (who did not report depression but reported at least one of the other coded diagnoses) and 1652 (49%) healthy controls (who did not report any diagnoses). In the depression group, comorbid mood and anxiety disorders were common – including anxiety and panic disorders (59.3%), obsessive–compulsive disorder (7.7%) and bipolar affective disorder (6.6%) – as well as personality disorders (12.7%) and psychotic disorders (12%). In the active controls group, the most commonly reported diagnoses were mood and anxiety disorders other than depression (35.5%), psychotic disorders (31%) and organic disorders (28.5%). Supplementary Table 1 available at https://doi.org/10.1192/bjo.2023.68 provides a full breakdown of the reported diagnoses.

Table 1 provides a summary of sociodemographic characteristics. The variable sample sizes are due to missing values, as detailed in Table 1. The depression group's median age was significantly lower than that of the active controls but not the healthy controls. The depression group had a significantly lower proportion of females than the active controls group but not the healthy controls. The depression group also had a significantly higher proportion of White participants than the healthy controls group but not the active controls group.

**Table 1:** Sociodemographic characteristics for the total population (*n* = 3345)^a^

				Effect of group		Depression *v*. active controls		Depression *v*. healthy controls	
	Depression group	Active controls	Healthy controls	Comparator test	Effect size (95% CI)	Comparator test	Effect size (95% CI)	Comparator test	Effect size (95% CI)
Age, years									
*n*	726	934	1595						
Mean (s.d.)	45.9 (16.7)	50.7 (18.9)	45.8 (17.9)						
Median (IQR)	46 (24)	50 (30)	45 (28)	*H* = 40.39^b^ *P* < 0.01^b^	0.02 (0.01–0.02)^c^	*U* = 293682^f^	0.02 (0.01,0.03)^c^	*U* = 569183^f^	0.00 (0.00–0.02)^c^
						*Z* = −4.68^f^		*Z* = −0.66^f^	
						*P* < 0.01^f^		*P* = 0.51^f^	
Gender, *n* (%)^d^									
*n*	737 (100)	952 (100)	1644 (100)						
Female	514 (70)	501 (53)	1166 (71)						
Male	223 (30)	451 (47)	478 (29)	*P* < 0.01^d^	0.17^e^	*P* < 0.01^d^	0.17^e^	*P* = 0.56^d^	0.01^e^
Ethnicity, *n* (%)									
*n*	736 (100)	953 (100)	1634 (100)						
White	694 (94)	876 (92)	1446 (89)						
Asian	13 (2)	40 (4)	98 (6)						
Black/African/Caribbean	10 (1)	17 (2)	45 (3)						
Mixed	11 (2)	10 (1)	25 (1)						
Other	8 (1)	10 (1)	20 (1)	*P* < 0.01^d^	0.66^e^	*P* = 0.06^d^	0.07^e^	*P* < 0.01^d^	0.10^e^

a. Totals in the table do not add up to *n* = 3345 because of missing data.

b. Kruskal–Wallis.

c. Eta-squared; effect sizes: small (0.01), medium (0.06) and large (0.14).

d. Chi-squared.

e. Cramér's *V*; effect sizes: small (0.1), medium (0.3) and large (0.5).

f. Mann–Whitney.

Table 2 provides a summary of descriptive statistics for each group for clinical severity (CGI score), fatigue (VAS-F score), ANS function (COMPASS-31 total weighted score) and cognitive function (THINC-it components), as well as between-group comparisons for the same measures. CGI score in the depression group was significantly higher than in healthy controls but comparable to that in active controls. The depression group, when compared with both control groups, had significantly higher median fatigue severity on the VAS-F. The depression group had significantly (*P* < 0.01) more severe autonomic dysregulation as measured by COMPASS-31 scores (median 30) than active (median 23) and healthy controls (median 10). The optional cognitive assessment with the THINC-it was completed by 499 participants (15% of the total sample). Significantly fewer participants in the depression group, when compared with both control groups, completed the THINC-it. The severity of subjective symptomatology on the PDQ-5 was significantly greater in the depression group than in both control groups. Participants in the depression group also scored significantly worse on objective measures, including both components of the DSST (higher average reaction time and lower total number of items correct), when compared with the active control group, and significantly worse on all objective components of the THINC-it except for the *N*-back average reaction time when compared with healthy controls.

**Table 2:** Measures of disease severity, fatigue, autonomic nervous system function and cognitive function, with between-group comparisons

		Depression group	Active controls	Healthy controls	Effect of group		Depression *v*. active controls		Depression *v*. healthy controls	
					Kruskal–Wallis	Effect size (95% CI)^a^	Mann–Whitney	Effect size (95% CI)^a^	Mann–Whitney	Effect size (95% CI)^a^
Clinical severity (CGI score), *n*, median (IQR)		375, 3 (2)	583, 3 (1)	217, 1 (0)	*H* = 313.70 *P* < 0.01	0.26 (0.22–0.30)	*U* = 102 768, *Z* = −1.61, *P* = 0.11	0.00 (0.00–0.01)	*U* = 11 878, *Z* = 15.13, *P* < 0.01	0.33 (0.27–0.39)
Fatigue (VAS-F score), *n*, median (IQR)		739, 60 (53)	951, 50 (55)	1650, 24 (46)	*H* = 278.89 *P* < 0.01	0.09 (0.07–0.11)	*U* = 299 047, *Z* = −5.27, *P* < 0.01	0.02 (0.01–0.03)	*U* = 369 228, *Z* = −15.45, *P* < 0.01	0.11 (0.09–0.13)
ANS function (COMPASS-31 total weighted score), *n*, median (IQR)		740, 30 (24)	953, 23 (25)	1652, 10 (17)	*H* = 635.66 *P* < 0.01	0.20 (0.17–0.22)	*U* = 243 407, *Z* = −7.28, *P* < 0.01	0.03 (0.02–0.05)	*U* = 238 102.5, *Z* = −21.99, *P* < 0.01	0.23 (0.20–0.26)
Cognitive function (THINC-it)										
*n*		127	119	253						
PDQ-5, median (IQR)	Total score	11 (8)	8 (8)	4 (4)	*H* = 129.97, *P* < 0.01	0.26 (0.20–0.32)	*U* = 6160.5, *Z* = −3.86, *P* < 0.01	0.06 (0.02–0.12)	*U* = 6005.5, *Z* = −10.56, *P* < 0.01	0.33 (0.26–0.40)
CRT, median (IQR)	Average reaction time	754 (474)	849 (445)	642 (343)	*H* = 39.17, *P* < 0.01	0.08 (0.04–0.12)	*U* = 7414, *Z* = 1.80, *P* = 0.07	0.02 (0.00–0.05)	*U* = 13 053.5, *Z* = −3.83, *P* < 0.01	0.04 (0.01–0.09)
	Total correct	36 (8)	36 (9)	38 (4)	*H* = 40.34 *P* < 0.01	0.07 (0.03–0.11)	*U* = 7680.5, *Z* = −1.37, *P* = 0.17	0.01 (0.00–0.04)	*U* = 12 498, *Z* = 4.45, *P* < 0.01	0.04 (0.01–0.09)
*N*-back, median (IQR)	Average reaction time	987 (274)	1019 (342)	965 (288)	*H* = 1.76 *P* = 0.42	0.00 (0.00–0.02)	*U* = 7475, *Z* = −0.26, *P* = 0.79	0.00 (0.00–0.02)	*U* = 15 151, *Z* = −1.02, *P* = 0.31	0.00 (0.00–0.02)
	Total correct	17 (18)	14 (15)	22 (19)	*H* = 44.19 *P* < 0.01	0.09 (0.04–0.13)	*U* = 7437.5, *Z* = −1.76, *P* = 0.08	0.01 (0.00–0.05)	*U* = 12 679.5, *Z* = −4.23, *P* < 0.01	0.05 (0.01–0.09)
DSST, median (IQR)	Average reaction time	2343 (1777)	2838 (2462)	2085 (906)	*H* = 57.75, *P* < 0.01	0.07 (0.03–0.11)	*U* = 6767, *Z* = −2.77, *P* < 0.01	0.02 (0.00–0.06)	*U* = 12 468.5, *Z* = −4.38, *P* < 0.01	0.04 (0.01–0.08)
	Total correct	43 (31)	34 (30)	52 (25)	*H* = 57.38, *P* < 0.01	0.11 (0.06–0.16)	*U* = 6674.5, *Z* = −3.01, *P* < 0.01	0.04 (0.01–0.09)	*U* = 12 697, *Z* = −4.21, *P* < 0.01	0.05 (0.01–0.09)
TMT-B, median (IQR)	Total time	34 (33)	44 (51)	26 (17)	*H* = 48.85, *P* < 0.01	0.09 (0.04–0.13)	*U* = 7407.5, *Z* = −1.81, *P* = 0.07	0.02 (0.00–0.07)	*U* = 11866, *Z* = −4.99, *P* < 0.01	0.05 (0.02–0.10)
	Number of errors	1 (2)	1 (3)	0 (1)	*H* = 21.36 *P* < 0.01	0.05 (0.02–0.09)	*U* = 7571.5, *Z* = −1.61, *P* = 0.11	0.01 (0.00–0.05)	*U* = 14 528, *Z* = −2.69, *P* < 0.01	0.03 (0.00–0.07)

ANS, autonomic nervous system; CGI, Clinical Global Impression – Severity scale; COMPASS-31, Composite Autonomic Symptom Scale-31; CRT, choice reaction time; DSST, Digit Symbol Substitution Test; PDQ-5, five-item Perceived Deficits Questionnaire; TMT-B, Part B of the Trail Making Test; VAS-F, Visual Analogue Scale for Fatigue.

a. Eta-squared; effect sizes: small (0.01), medium (0.06) and large (0.14).

**Fig. 1:**
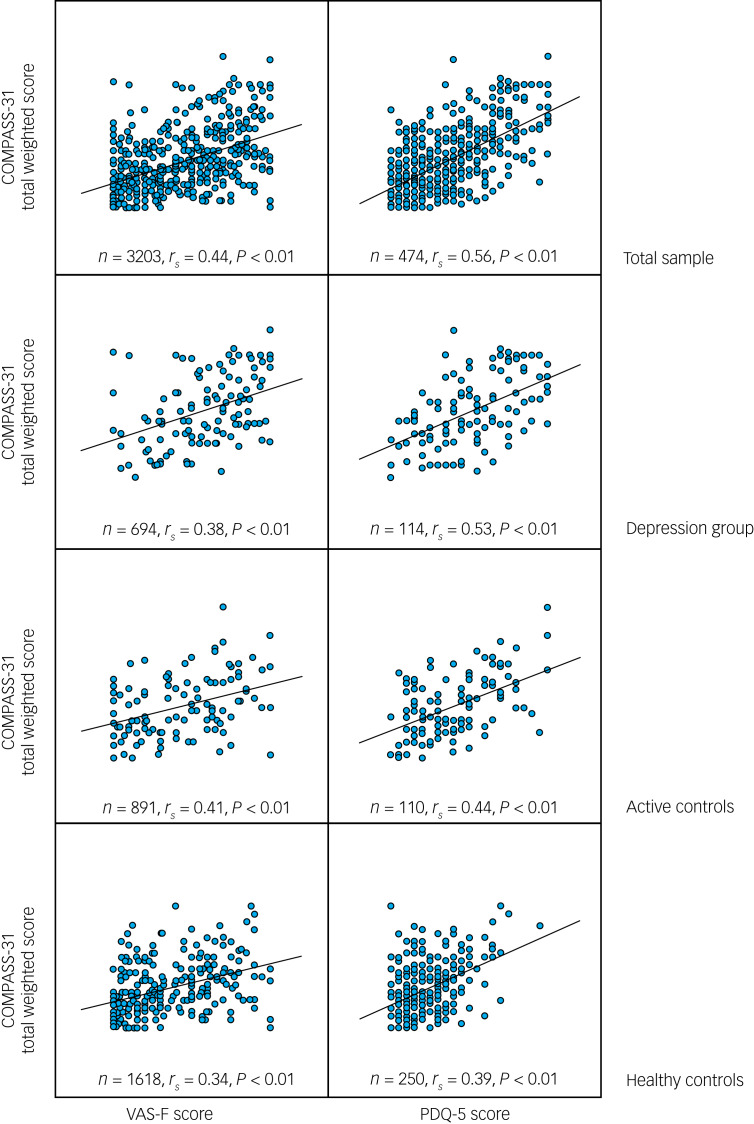
Scatter plots between COMPASS-31 total weighted score, VAS-F scores and PDQ-5 scores. COMPASS-31: Composite Autonomic Symptom Scale-31; PDQ-5, five-item Perceived Deficits Questionnaire; VAS-F, Visual Analogue Scale for Fatigue. Spearman's rho (r_s_), 2-tailed; effect sizes: small (0.1), medium (0.3) and large (0.5).

Table 3 provides a summary of the correlations between measures of ANS function (COMPASS-31 total weighted score) and measures of clinical severity (CGI score), fatigue (VAS-F score) and cognitive function (THINC-it components). Scatter plots between COMPASS-31 total weighted score, VAS-F scores and PDQ-5 scores are shown in Fig. 1. The relationship between COMPASS-31 and both the VAS-F and the PDQ-5 had a Spearman correlation coefficient of ≥0.34 (*P* < 0.01) for the total sample and the three individual groups (depression, active controls and healthy controls). This relationship was not seen for the THINC-it objective cognitive measures for any of the participant individual groups. Supplementary Table 2 provides a summary of the results for the hierarchical multiple regression analyses for variables predicting objective THINC-it measures (the dependent variables) using COMPASS-31 scores and group membership. The hierarchical multiple regression models were significant for all measures except *N*-back average reaction time. The models predicted a small amount of the variation in objective THINC-it measures (3–7%).

**Table 3:** Spearman correlation (2-tailed) between autonomic nervous system function (COMPASS-31 total weighted score) and measures of disease severity, fatigue and cognitive function

			Depression group	Active controls	Healthy controls	Total sample
Clinical severity (CGI score)						
*n*			341	547	209	1097
*r_s_^a^*			0.11	0.08	0.17	0.23
*P*			0.04	0.06	0.02	<0.01
Fatigue (VAS-F score)						
*n*			694	891	1618	3203
*r_s_*^a^			0.38	0.41	0.34	0.44
*P*			<0.01	<0.01	<0.01	<0.01
Cognitive function (THINC-it)						
*n*			114	110	250	474
PDQ-5	Total score	*r_s_*	0.53	0.44	0.39	0.56
		*P*	<0.01	<0.01	<0.01	<0.01
CRT	Average reaction time	*r_s_*	0.09	0.13	0.11	0.17
		*P*	0.29	0.17	0.09	<0.01
	Total correct	*r_s_*	−0.20	−0.20	−0.09	−0.22
		*P*	0.03	0.03	0.17	<0.01
*N*-back	Average reaction time	*r_s_*	−0.04	−0.06	−0.07	−0.05
		*P*	0.65	0.56	0.26	0.29
	Total correct	*r_s_*	−0.07	0.07	−0.03	−0.12
		*P*	0.49	0.48	0.60	<0.01
DSST	Average reaction time	*r_s_*	0.03	0.04	0.05	0.16
		*P*	0.78	0.66	0.43	<0.01
	Total correct	*r_s_*	−0.03	−0.03	−0.07	−0.16
		*P*	0.75	0.79	0.24	<0.01
TMT-B	Total time	*r_s_*	−0.04	0.03	0.05	0.13
		*P*	0.65	0.79	0.40	<0.01
	Number of errors	*r_s_*	0.09	−0.02	0.05	0.10
		*P*	0.33	0.85	0.48	0.03

CGI, clinical Global Impression – Severity scale; COMPASS-31, Composite Autonomic Symptom Scale-31; CRT, choice reaction time; DSST, Digit Symbol Substitution Test; PDQ-5, five-item Perceived Deficits Questionnaire; TMT-B, Part B of the Trail Making Test; VAS-F, Visual Analogue Scale for Fatigue.

a. Spearman's rho (2-tailed); effect sizes: small (0.1), medium (0.3) and large (0.5).

**Fig. 2:**
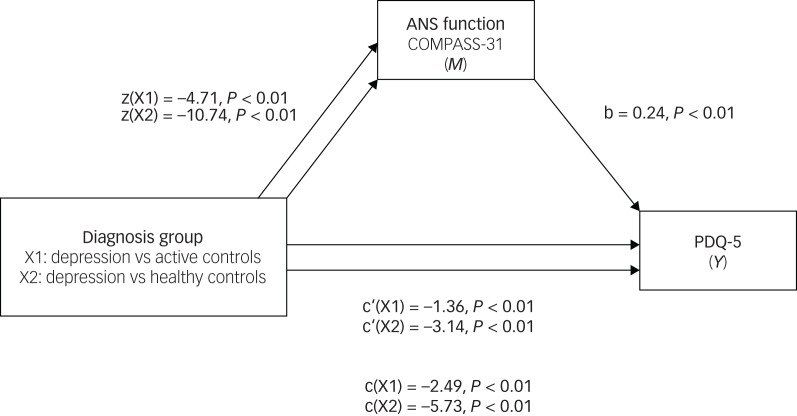
Mediation analysis: standardised regression coefficients for the relationship between diagnosis group and score on the PDQ-5, as mediated by COMPASS-31 scores. ANS, autonomic nervous system; COMPASS-31, Composite Autonomic Symptom Scale-31; PDQ-5, five-item Perceived Deficits Questionnaire.

**Fig. 3:**
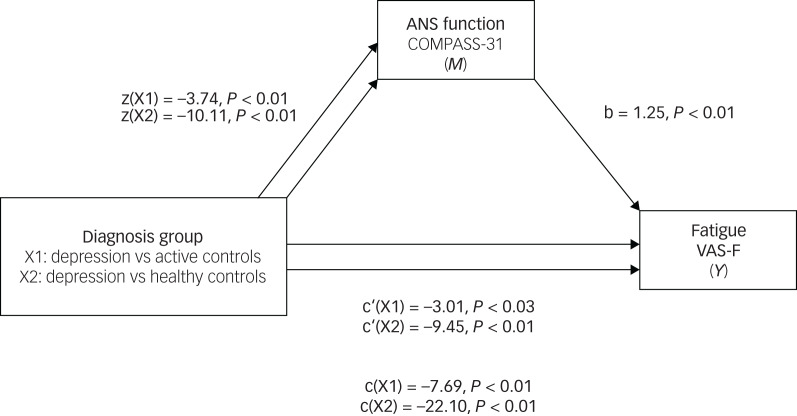
Mediation analysis: standardised regression coefficients for the relationship between diagnosis group and VAS-F scores, as mediated by COMPASS-31 scores. ANS, autonomic nervous system; COMPASS-31, Composite Autonomic Symptom Scale-31; VAS-F, Visual Analogue Scale for Fatigue.

Mediation models were used to investigate the hypothesis that ANS function mediates the effects of diagnosis on participants’ subjective assessment of their cognition and fatigue (Figs 2, 3). A distinct model was constructed for each dependent variable. ANS function was a significant predictor of PDQ-5 scores, both when comparing the depression group with active controls (*B* = −1.13, s.e. = 0.34, 95% CI −1.85 to −0.51) and with healthy controls (*B* = −2.58, s.e. = 0.35, 95% CI −3.29 to −1.93). When considering the total effect (path c) of diagnostic group predicting PDQ-5 scores as mediated by ANS function, on average the depression group scored 2 points higher (worse) than active controls (*b* = −2.49, *t*(465) = −4.33, *P* < 0.01) and 6 points higher (worse) than healthy controls (*b* = −5.73, *t*(465) = −11.76, *P* < 0.01). ANS function was a significant predictor of fatigue scores, both when comparing the depression group with active controls (*B* = −4.68, s.e. = 0.69, 95% CI −6.02 to −3.34) and with healthy controls (*B* = −12.65, s.e. = 0.76, 95% CI −14.15 to −11.15). When considering the total effect (path c) of diagnostic group predicting fatigue scores as mediated by ANS function, on average, the depression group scored 8 points higher (worse) than active controls (*b* = −7.69, *t*(3163) = −5.10, *P* < 0.01) and 22 points higher (worse) than healthy controls (*b* = −22.10, *t*(3163) = −16.38, *P* < 0.01). These results support the hypothesis that ANS function (measured by COMPASS-31 scores) may partially mediate the effects of a depression diagnosis on PDQ-5 and VAS-F scores.

Supplementary Table 3 provides a summary of prescribed medication. Overall, 1201 (36%) participants were prescribed one or more psychotropic drugs. Of the three groups, the depression group had the highest proportion of psychotropic prescription (76%). Just 4% of the healthy controls group reported using any psychotropic.

Table 4 provides a summary of COMPASS-31 total weighted scores for each group, according to medication prescribed, as well as paired between-group comparisons. Participants prescribed medication (1140), when compared with those not prescribed (2063), had significantly greater ANS dysfunction on the COMPASS-31. In the group of participants with depression, there was no significant difference between ANS function in those prescribed psychotropics (specifically, antidepressants, antipsychotics and mood stabilisers) compared with those not prescribed any psychotropics. Overall, participants prescribed anticholinergics, when compared with those not prescribed anticholinergics, had significantly greater ANS dysfunction. Overall, participants prescribed beta-blockers, when compared with those not prescribed beta-blockers, had significantly greater ANS dysfunction. When comparing for all prescribed medication groups, COMPASS-31 total weighted scores remained significantly different between the depression group, active controls and healthy controls, with variable effect sizes. Differences were significant for all paired between-group comparisons, except between the depression group and active controls prescribed antidepressants and mood stabilisers, and the depression group and healthy controls prescribed antipsychotics.

**Table 4:** Measures of autonomic nervous system function (COMPASS-31 total weighted score) for each group according to prescribed medication, including between-group comparisons

COMPASS-31 total weighted score		Depression group	Active controls	Healthy controls	Depression *v*. active controls *v*. healthy controls		Depression *v*. active controls		Depression *v*. healthy controls	
					*P*^a^	Effect size^b^ (95% CI)	*P*^c^	Effect size^b^ (95% CI)	*P*^c^	Effect size^b^ (95% CI)
**Any psychotropic**										
Prescribed	*n*	527	544	69						
	Median (IQR)	21 (16)	18 (16)	13 (14)	<0.01	0.02 (0.01–0.04)	<0.01	0.02 (0.00–0.03)	<0.01	0.03 (0.01–0.07)
Not prescribed	*n*	167	347	1549						
	Median (IQR)	20 (16)	14 (13)	9 (10)	<0.01	0.11 (0.09–0.14)	<0.01	0.05 (0.02–0.09)	<0.01	0.12 (0.09–0.14)
Prescribed *v*. Not prescribed	*P*^c^	0.31	<0.01	<0.01						
	Effect size^d^ (95% CI)	0.12 (0.05–0.30)	0.33 (0.19–0.46)	0.60 (0.36–0.85)						
**Antidepressants**										
Prescribed	*n*	461	249	52						
	Median (IQR)	21 (16)	18 (15)	14 (17)	<0.01	0.02 (0.01–0.04)	0.06	0.00 (0.00–0.02)	<0.01	0.02 (0.00–0.05)
Not prescribed	*n*	233	642	1566						
	Median (IQR)	20 (16)	15 (15)	9 (10)	<0.01	0.14 (0.12–0.17)	<0.01	0.04 (0.02–0.06)	<0.01	0.15 (0.13–0.18)
Prescribed *v*. Not prescribed	*P*^c^	0.63	<0.01	<0.01						
	Effect size^d^ (95% CI)	0.03 (−0.13 to 0.18)	0.33 (0.18–0.47)	0.70 (0.42–0.97)						
**Antipsychotics**										
Prescribed	*n*	166	367	11						
	Median (IQR)	23 (15)	18 (16)	11 (21)	<0.01	0.03 (0.01–0.06)	<0.01	0.03 (0.01–0.06)	0.07	0.02 (0.00–0.07)
Not prescribed	*n*	528	524	1607						
	Median (IQR)	20 (15)	15 (14)	9 (10)	<0.01	0.17 (0.15–0.20)	<0.01	0.04 (0.02–0.06)	<0.01	0.20 (0.17– 0.23)
Prescribed *v*. Not prescribed	*P*^c^	0.07	<0.01	0.20						
	Effect size^d^ (95% CI)	0.18 (0.01–0.35)	0.18 (0.05–0.32)	0.75 (0.16–1.35)						
**Mood stabilisers**										
Prescribed	*n*	56	125	4						
	Median (IQR)	21 (15)	18 (15)	6 (10)	0.03	0.04 (0.00–0.96)	0–29	0.01 (0.00–0.06)	<0.01	0.09 (0.00–0.25)
Not prescribed	*n*	638	766	1614						
	Median (IQR)	21 (16)	15 (15)	9 (10)	<0.01	0.18 (0.15–0.20)	<0.01	0.03 (0.02–0.05)	<0.01	0.22 (0.20–0.25)
Prescribed *v*. Not prescribed	*P*^c^	0.78	0.04	0.42						
	Effect size^d^ (95% CI)	0.02 (−0.26 to 0.29)	0.15 (−0.04 to 0.34)	0.40 (−0.58 to 1.38)						
**Sedatives and anxiolytics**										
Prescribed	*n*	148	158	16						
	Median (IQR)	25 (17)	20 (20)	11 (14)	<0.01	0.06 (0.02–0.12)	<0.01	0.03 (0.00–0.07)	<0.01	0.11 (0.03–0.20)
Not prescribed	*n*	546	733	1602						
	Median (IQR)	19 (15)	15 (14)	9 (10)	<0.01	0.15 (0.13–0.17)	<0.01	0.03 (0.01–0.05)	<0.01	0.18 (0.16–0.21)
Prescribed *v*. Not prescribed	*P*^c^	<0.01	<0.01	0.65						
	Effect size^d^ (95% CI)	0.55 (0.36–0.73)	0.50 (0.32–0.67)	0.23 (0.26–0.72)						
**Anticholinergics**										
Prescribed	*n*	127	202	27						
	Median (IQR)	22 (17)	17 (15)	16 (18)	<0.01	0.04 (0.01–0.08)	<0.01	0.04 (0.01–0.08)	0.02	0.04 (0.00–0.11)
Not prescribed	*n*	567	689	1591						
	Median (IQR)	20 (15)	16 (15)	9 (10)	<0.01	0.18 (0.15–0.20)	<0.01	0.03 (0.01–0.05)	<0.01	0.22 (0.19–0.25)
Prescribed *v*. Not prescribed	*P*^c^	0.22	0.25	<0.01						
	Effect size^d^ (95% CI)	0.14 (−0.06 to 0.33)	0.08 (−0.08 to 0.24)	0.77 (0.39–1.16)						
**Beta-blockers**										
Prescribed	*n*	41	72	34						
	Median (IQR)	22 (19)	14 (12)	10 (13)	<0.01	0.12 (0.04–0.22)	0.02	0.05 (0.00–0.15)	<0.01	0.21 (0.07–0.36)
Not prescribed	*n*	653	819	1584						
	Median (IQR)	21 (15)	16 (15)	9 (10)	<0.01	0.18 (0.15–0.20)	<0.01	0.03 (0.01–0.05)	<0.01	0.23 (0.20–0.26)
Prescribed *v*. Not prescribed	*P*^c^	0.84	0.54	0.69						
	Effect size^d^ (95% CI)	0.07 (−0.25 to 0.39)	0.09 (−0.15 to 0.33)	0.05 (−0.29 to 0.39)						

Data are presented for each group (depression, active controls and healthy controls) on the number of participants prescribed and not prescribed the respective drug class (*n*), as well as the median and IQR for COMPASS-31 in each group. All between-group comparisons are for COMPASS-31 scores. In the rows, ‘Prescribed *v*. Not prescribed’ compares the COMPASS-31 scores for those prescribed and not prescribed the respective drug class. In the columns, comparisons in COMPASS-31 scores are presented between the three groups using the Kruskal–Wallis test (‘depression *v*. active controls *v*. healthy controls’) and pairwise using the Mann–Whitney test (‘depression *v*. active controls’ and ‘depression *v*. healthy controls’).

a. Kruskal–Wallis.

b. Eta-squared; effect sizes: small (0.01), medium (0.06) and large (0.14).

c. Mann–Whitney.

d. Cohen's d; effect sizes: small (0.2), medium (0.5) and large (0.8).

## Discussion

This study aimed to examine the relationship between self-reported ANS symptoms, fatigue, cognitive function, clinical severity and prescribed medication in people with depression and in comparators with and without other mental health, neurodevelopmental or neurodegenerative disorders. Our results show that people with psychiatric diagnoses have greater self-reported autonomic dysregulation symptoms, levels of fatigue and cognitive deficits. The effects of depression and other disorders on fatigue and self-perceived cognitive deficits appear to be mediated, at least in part, by ANS dysregulation. Prescribed medication does not fully explain the differences in ANS dysregulation scores.

The self-reported scores for ANS function (COMPASS-31), fatigue (VAS-F) and self-perceived cognitive deficits (PDQ-5) showed that the depression group had significantly and consistently more severe automatic dysregulation than both the active and healthy controls groups. Effect sizes were small when comparing the depression group with active controls. It is notable that the depression group had consistently and significantly more severe dysregulation, even though active controls were a heterogeneous group with a range of diagnoses. On the other hand, effect sizes were medium to large when comparing the depression group with healthy controls. These effect sizes were particularly large (with narrow confidence intervals) when comparing ANS symptoms and self-perceived cognitive deficits. Clinical severity, as measured by the CGI, was similar in the depression and active controls groups. Unsurprisingly, the CGI scores for the healthy controls were significantly lower (the median score was 1, the lowest possible). Overall, this is reassuring as regards the robustness of the group classification in the sample, particularly the classification of ‘healthy’ controls, despite diagnoses being self-rated, and consistent with the very low rate (4%) of psychotropic use in this group.

ANS dysregulation in depression and other psychiatric disorders has been widely reported, including meta-analytic evidence that HRV (as a proxy for ANS function), when compared with controls, is reduced in people with mood, psychotic and substance dependence disorders even if they are not prescribed medication.^8^ The underlying mechanisms explaining these associations are not clear. Resolution of depressive symptoms does not appear to consistently lead to HRV normalisation, suggesting that HRV might be a trait rather than state biomarker.^16^ There is limited published data on autonomic symptoms in people with depression and other psychiatric disorders. To put our results in perspective, the median COMPASS-31 scores in our sample (30 and 23 for the depression and active controls groups respectively) are more severe than those of a small sample of patients with mild cognitive impairment with Lewy bodies (median 12.5).^6^

The THINC-it has been demonstrated to be valid for the cognitive assessment of people with depression^23^ and healthy controls.^24^ The PDQ-5, derived from the Perceived Deficits Questionnaire (PDQ),^25^ is the only self-report component of the THINC-it. Considering the whole subsample who completed the THINC-it (474 participants), COMPASS-31 scores positively and significantly correlated with more severe self-perceived cognitive deficits. The PDQ-5 scores in the depression group of our sample (median 11, IQR = 8) were similar to baseline scores from a large prospective European multicentre study involving patients with major depression (median 12, IQR = 7).^26^ The depression group scored on average 6 points higher (worse) than healthy controls in the mediation analysis, and the deficits appear to be due, at least in part, to the autonomic dysfunction. PDQ-5 scores have previously been shown to be a significant prospective determinant of functional impairment and overall depression severity in people with major depression.^26^ For the objective cognitive tasks, the differences in scores between the depression group and active controls were statistically significant only for the DSST (with small effect sizes), a task which is affected by psychomotor speed, fatigue and ageing.^27,28^ When comparing the depression group with healthy controls, results on all objective measures of the THINC-it were significantly worse (with small to medium effect sizes), except for the average reaction time on the *N*-back, a separation which has not been seen in previous smaller studies.^20^

COMPASS-31 scores positively and significantly correlated with more severe levels of fatigue (VAS-F), having medium effect sizes across the three groups. This significant association was also found in the mediation analysis, where the depression group scored on average 22 points higher (worse) on the VAS-F than healthy controls. This correlation was mediated, at least in part, by COMPASS-31 scores. This is a clinically significant difference: the minimal important difference for global change on the VAS-F is estimated to range from 7 to 17.^29^ This mirrors the demonstration of a correlation between objective and subjective measures of ANS dysregulation with fatigue in other populations,^3,5,12^ suggesting a transdiagnostic impact of ANS dysregulation.

Psychotropic medication is known to affect both autonomic^8^ and cognitive^10^ functions. Antidepressant prescription, particularly of tricyclics, has been associated with decreases in cardiac vagal control as measured by HRV in longitudinal^30^ and case–control studies.^31^ Therefore, it is pertinent to consider whether psychotropic use may explain the study findings. ANS dysfunction remained significantly worse in the depression group in both participants prescribed and not prescribed psychotropics. Further, in this group COMPASS-31 scores did not significantly differ with prescription of antidepressants, antipsychotics or mood stabilisers. Tricyclics, known to have significant cholinergic burden as a class represented only 7% of the medication prescribed, limiting power for more detailed analysis. Overall, meta-analytic evidence on the relationship between objective measures of ANS dysregulation (such as HRV) and psychiatric disorders^8^ aligns with our findings: HRV is reduced in patients with mood and psychotic disorders compared with controls, and the associations remain significant in people not prescribed medication. In summary, our results support the notion that ANS dysregulation, indexed by COMPASS-31 scores, is associated with depression and that this relationship is not fully explained by the prescribed medication.

This study has shown correlation between several self-report measures, including measures relating to symptoms putatively related to ANS dysregulation, fatigue and cognitive function. This raises a number of questions. Are these distinct and independent constructs or might they overlap? The COMPASS questionnaire, for instance, contains a question about sleep, and ‘fatigue’ might imply a mental as well as a physical phenomenon.^32^ One also wonders about the impact of the halo effect and, relatedly, whether an individual's response tendencies may be important: negative cognitions in people with depression might make them more likely to self-report worse scores relative to those that they obtained in objective tests. There are examples in the literature, for instance, of depression scale scores correlating with seemingly related (e.g. PDQ in multiple sclerosis^33^) and seemingly unrelated (e.g. dryness in Sjögren's syndrome^34^) symptoms.

### Limitations

This study has several limitations. We used no formal criteria for study inclusion or diagnoses definition. The self-reported diagnoses were used for study group definition, which was central to our methods. Even if group classification was reliable, those who consented to participate might not be representative of all patients with mental, neurodevelopmental and neurodegenerative disorders. They could represent a group of people with higher burden or, conversely, a group more motivated to participate and less fatigued. Any participant who reported depression was included in the ‘depression’ group for this analysis, regardless of comorbidities. Although this highlights the relevance of considering multimorbidity when studying people with depression, it also makes the ‘depression’ group heterogeneous. We considered and reported comorbid diagnoses (Supplementary Table 1) but did not control for them, as most of the diagnostic ‘subgroups’ are small and this would limit power. Health-related behaviours such as smoking, alcohol use, exercise levels, quality of sleep and diet were not explored. Cognitive measures were available only for a subsample and, again, this could represent a distinct subgroup. Trial runs of the THINC-it were not controlled for, and this is of relevance, as there will be a practice effect. The THINC-it was delivered using a variety of input methods (such as keyboards and touch screens), which will affect reaction times, and this was not controlled for. Although the overall sample is relatively large, some subsets (such as the number of patients prescribed tricyclics or betablockers) are small and limit power. Our correlational analysis between COMPASS-31 scores and prescribed medication did not discriminate between antidepressant group (such as SSRIs, serotonin–noradrenaline reuptake inhibitors (SNRIs) and TCAs) and these are known to have distinct effects on HRV.^16^

### Further research

Many fundamental questions remain unanswered. For example, our methodology does not allow us to make any inferences on causality. There is now a need to understand the underlying mechanisms and the directionality of the associations. An important piece of work would be to untangle which changes to ANS function are related to the underlying diagnosis and which are driven by the effects of medication. This can only be achieved with controlled trials (for example, measuring ANS function before and after medication or neurostimulation) or even with a prospective longitudinal cohort that captures population level data prior to diagnosis and at several time points onwards. Given the high prevalence of depression in the general population, this would be feasible. These studies could be important to exploring the pathophysiology of depression and better understanding what drives sensibility to cholinergic side-effects. Another step would be to combine self-reported rating scales and objective physiological measures. This would be relevant to correlating symptoms of ANS dysregulation with biomarkers (such as HRV), which could allow us to better monitor response and personalise treatment. Fatigue is a core and burdensome symptom of depression whose pathophysiology is not clear. Cognitive dysfunction associated with depression is known to be an area of unmet clinic need.^35^ It is important to clarify in controlled studies how modifying fatigue and cognitive performance changes biomarkers that shed light on mechanistic hypotheses – such as those related to ANS function – and how that correlates with overall quality of life. With clear evidence of mechanistic relationships between fatigue, cognition and ANS dysregulation, as is suggested by our cross-sectional data, novel treatment approaches could be designed targeting the ANS directly.

### Clinical implications

People with psychiatric diagnoses have more severe self-rated autonomic dysfunction, levels of fatigue and self-perceived cognitive deficits. The effects of depression and other disorders on fatigue and self-perceived cognitive deficits appear to be mediated by ANS dysregulation. If this is the case, treatments targeted at autonomic dysregulation could be helpful in reducing the burden of symptoms of cognitive impairments and fatigue in patients with mental disorders such as depression.

## Data Availability

The data that support the findings of this study are available from the corresponding author, T.C., on reasonable request

## Supplementary material

For supplementary material accompanying this paper visit http://doi.org/10.1192/bjo.2023.68. click here to view supplementary material
