# Supplementary material for: Autonomic dysregulation, cognition and fatigue in people with depression and in active and healthy controls: observational cohort study
Source: BJPsych Open. 2023 Jun 14;9(4):e106. doi: 10.1192/bjo.2023.68 (PMC10305035; doi:10.1192/bjo.2023.68)
Supplement: Supplementary file 1 [file S2056472423000686sup001.docx]

**SUPPLEMENTARY MATERIAL**

CAPMEM Paper – BJPsych Open

**Supplementary Table 1.** Diagnosis reported

| **Diagnosis**^a^ | **Depression**  **group**  n (%) | | **Active controls**  **group**  n (%) |
| --- | --- | --- | --- |
| Organic disorders | 56 (7.6) | 272 (28.5) | |
| Dementia | 23 (3.1) | 139 (14.6) | |
| Mild cognitive impairment | 6 (0.8) | 51 (5.4) | |
| Other neurodevelopmental disorders | 29 (3.9) | 98 (10.3) | |
| Disorders due to psychoactive substance use | 43 (5.8) | 37 (3.9) | |
| Alcohol use disorder | 35 (4.7) | 26 (2.7) | |
| Other substance use disorders | 16 (2.2) | 16 (1.7) | |
| Psychotic disorders | 89 (12) | 295 (31) | |
| Schizophrenia | 53 (7.2) | 186 (19.5) | |
| Other psychotic disorders | 42 (5.7) | 119 (12.5) | |
| Mood and anxiety disorders | 740 (100) | 338 (35.5) | |
| Depression | 740 (100) | 0 | |
| Bipolar affective disorder | 49 (6.6) | 153 (16.1) | |
| Generalized anxiety disorder | 211 (28.5) | 97 (10.2) | |
| Panic disorder | 78 (10.5) | 26 (2.7) | |
| Obsessive compulsive disorder | 57 (7.7) | 29 (3) | |
| Other anxiety disorders | 150 (20.3) | 94 (9.9) | |
| Personality disorders | 94 (12.7) | 70 (7.3) | |
| Borderline personality disorder | 76 (10.3) | 53 (5.6) | |
| Other personality disorders | 23 (3.1) | 17 (1.8) | |
| Neurodevelopment disorders | 28 (3.8) | 47 (4.9) | |
| Autism spectrum disorder | 19 (2.6) | 28 (2.9) | |
| Attention deficit hyperactivity disorder | 12 (1.6) | 21 (2.2) | |
| Other neurodevelopmental disorders | 0 | 4 (0.4) | |
| Eating disorders | 39 (5.3) | 24 (2.5) | |
| Other disorders^b^ | 55 (7.4) | 116 (12.2) | |
| Post-traumatic anxiety disorder^b^ | 33 (4.5) | 29 (3) | |
| Stroke^b^ | 2 (0.3) | 12 (1.3) | |
| Epilepsy^b^ | 5 (0.7) | 5 (0.5) | |
| Multiple sclerosis^b^ | 14 (1.9) | 62 (6.5) | |
| Parkinson’s disease^b^ | 3 (0.4) | 9 (0.9) | |

^a^ Participants can report multiple diagnosis, therefore sub-total in groups do not add up to 100%

^b^ Manually inputted from “free text” box

**Supplementary Table 2.** Hierarchical multiple regression analyses for variables predicting objective THINC-it measures (n=474)

| Variables | β | *t* | R | R^2^ | ∆R^2^ | p-value |
| --- | --- | --- | --- | --- | --- | --- |
| **Dependent variable: CRT average reaction time** | | | | | | |
| Step 1 |  |  | 0.18 | 0.03 |  | <0.01 |
| COMPASS-31 | 5.25 | 4.05 |  |  |  | <0.01 |
| Step 2 |  |  | 0.07 | 0.06 | 0.03 | <0.01 |
| Active controls | 57.73 | 1.54 |  |  |  | 0.12 |
| Healthy controls | -82.39 | -2.33 |  |  |  | 0.02 |
| **Dependent variable: CRT total correct** | | | | | | |
| Step 1 |  |  | 0.20 | 0.04 |  | <0.01 |
| COMPASS-31 | -0.14 | -4.54 |  |  |  | <0.01 |
| Step 2 |  |  | 0.27 | 0.08 | 0.03 | <0.01 |
| Active controls | -2.04 | -2.24 |  |  |  | 0.03 |
| Healthy controls | 1.23 | 1.43 |  |  |  | 0.15 |
| **Dependent variable:** **N-Back average reaction time** | | | | | | |
| Step 1 |  |  | 0.04 | 0.00 |  | 0.46 |
| COMPASS-31 | -0.84 | -0.74 |  |  |  | 0.46 |
| Step 2 |  |  | 0.06 | 0.00 | 0.00 | 0.54 |
| Active controls | 7.62 | 0.23 |  |  |  | 0.82 |
| Healthy controls | -23.21 | -0.74 |  |  |  | 0.45 |
| **Dependent variable: N-Back total correct** | | | | | | |
| Step 1 |  |  | 0.14 | 0.02 |  | <0.01 |
| COMPASS-31 | -0.16 | -3.05 |  |  |  | <0.01 |
| Step 2 |  |  | 0.28 | 0.08 | 0.06 | <0.01 |
| Active controls | -2.71 | -1.80 |  |  |  | 0.07 |
| Healthy controls | 4.25 | 2.99 |  |  |  | <0.01 |
| **Dependent variable: DSST average reaction time** | | | | | | |
| Step 1 |  |  | 0.09 | 0.01 |  | 0.05 |
| COMPASS-31 | 24.84 | 1.99 |  |  |  | 0.05 |
| Step 2 |  |  | 0.27 | 0.07 | 0.06 | <0.01 |
| Active controls | 1071.56 | 3.00 |  |  |  | <0.01 |
| Healthy controls | -711.43 | -2.12 |  |  |  | 0.03 |
| **Dependent variable: DSST total correct** | | | | | | |
| Step 1 |  |  | 0.16 | 0.03 |  | <0.01 |
| COMPASS-31 | -0.39 | -3.53 |  |  |  | <0.01 |
| Step 2 |  |  | 0.32 | 0.10 | 0.07 | <0.01 |
| Active controls | -8.41 | -2.66 |  |  |  | <0.01 |
| Healthy controls | 8.81 | 2.96 |  |  |  | <0.01 |
| **Dependent variable: TMT-B total time** | | | | | | |
| Step 1 |  |  | 0.10 | 0.01 |  | 0.03 |
| COMPASS-31 | 0.43 | 2.18 |  |  |  | 0.03 |
| Step 2 |  |  | 0.28 | 0.08 | 0.07 | <0.01 |
| Active controls | 12.73 | 2.28 |  |  |  | 0.02 |
| Healthy controls | -15.56 | -2.97 |  |  |  | <0.01 |
| **Dependent variable: TMT-B N of errors** | | | | | | |
| Step 1 |  |  | 0.11 | 0.01 |  | 0.02 |
| COMPASS-31 | 0.02 | 2.40 |  |  |  | 0.02 |
| Step 2 |  |  | 0.21 | 0.05 | 0.03 | <0.01 |
| Active controls | 0.53 | 1.88 |  |  |  | 0.06 |
| Healthy controls | -0.48 | -1.79 |  |  |  | 0.08 |

COMPASS-31: Composite Autonomic Symptom Scale-31; CRT: Choice Reaction Time; DSST: Digit Symbol Substitution Test; PDQ-5: 5-item Perceived Deficits Questionnaire; TMT-B: Part B of the Trial Making Test

**Supplementary Table 3.** Summary of prescribed medication

| **Prescribed medication** | | **Depression group**  n (%) | | **Active controls group**  n (%) | | **Healthy controls group**  n (%) | | **Total sample**  n (%) |
| --- | --- | --- | --- | --- | --- | --- | --- | --- |
| **Any psychotropic**^a^ | | 559 (76%) | | 571 (60%) | | 71 (4%) | | 1201 (36%) |
| **Antidepressants** | | 490 (66%) | | 263 (28%) | | 53 (3%) | | 806 (24%) |
| SSRIs^b^ | | 309 (42%) | | 178 (19%) | | 32 (2%) | | 519 (16%) |
| SNRIs^c^ | | 91 (12%) | 39 (4%) | | | 2 (<1%) | 132 (4%) | |
| TCAs^d^ | | 49 (7%) | 27 (3%) | | | 21 (1%) | 97 (3%) | |
| MAOI^e^ | | 1 (<1%) | 0 | | | 0 | 1 (<1%) | |
| NaSSA^f^ | | 78 (11%) | 33 (4%) | | | 3 (<1%) | 114 (3%) | |
| Other antidepressants^g^ | | 23 (3%) | 12 (1%) | | | 0 | 35 (1%) | |
| Unspecified “antidepressant” | | 4 (<1%) | 0 | | | 0 | 4 (<1%) | |
| **Antipsychotics** | | 178 (24%) | | 386 (41%) | | 12 (<1%) | | 576 (17%) |
| 1^st^ generation – “typicals”^h^ | | 18 (2%) | | 53 (6%) | | 1 (<1%) | | 72 (2%) |
| 2^nd^ generation – “atypicals”^i^ | | 165 (22%) | | 346 (36%) | | 11 (<1%) | | 522 (16%) |
| **Mood stabilizers** | | 62 (8%) | | 135 (14%) | | 6 (<1%) | | 203 (6%) |
| Carbamazepine |  | 5 (<1%) | | | 13 (1%) | 0 | | 18 (<1%) |
| Lamotrigine |  | 16 (2%) | | | 38 (4%) | 3 (<1%) | | 57 (2%) |
| Lithium |  | 22 (3%) | | | 40 (4%) | 0 | | 62 (2%) |
| Valproate |  | 20 (3%) | | | 62 (7%) | 3 (<1%) | | 85 (3%) |
| **Sedatives and anxiolytics** | | 153 (21%) | | | 164 (17%) | 17 (1%) | | 334 (10%) |
| Benzodiazepines |  | 71 (10%) | | | 97 (10%) | 6 (<1%) | | 174 (5%) |
| Zopiclone and zolpidem | | 44 (6%) | | | 45 (5%) | 3 (<1%) | | 92 (3%) |
| Gabapentin and pregabalin | | 66 (9%) | | | 48 (5%) | 10 (<1%) | | 124 (4%) |
| Promethazine |  | 27 (4%) | | | 34 (4%) | 4 (<1%) | | 65 (2%) |
| **Others** |  |  | | |  |  | |  |
| Anticholinergics^j^ | | 134 (18%) | | | 211 (22%) | 28 (2%) | | 373 (11%) |
| Beta-blockers |  | 46 (6%) | | | 76 (8%) | 36 (2%) | | 158 (5%) |

MAOI: monoamine oxidase inhibitor; NaSSA: noradrenergic and specific serotonergic antidepressants; SSRIs: selective serotonina reuptake inhibitors; SNRI: serotonin and norepinephrine reuptake inhibitors; TCAs: tricyclic antidepressants.

^a^ Prescribed any of the following: antidepressants, antipsychotics, mood stabilizers or sedatives and anxiolytics.

^b^ Including: citalopram, escitalopram, fluoxetine, fluvoxamine, paroxetine and sertraline.

^c^ Including: duloxetine and venlafaxine.

^d^ Including: amitriptyline, clomipramine, dosulepin, doxepin, lofepramine, nortriptyline and trimipramine

^e^ The only MAOI reported was moclobemide.

^f^ This group comprised only of mirtazapine.

^g^ Including: agomelatine, bupropion, reboxetine, trazodone and vortioxetine.

^h^ Including: chlorpromazine, flupentixol, haloperidol, levomepromazine, prochlorperazine, trifluoperazine and zuclopenthixol.

^i^ Including: amisulpride, aripiprazole, clozapine, lurasidone, olanzapine, paliperidone, quetiapine, risperidone and sulpiride.

^j^ The “anticholinergics” group includes paroxetine, all the TCAs, chlorpromazine, clozapine, olanzapine and promethazine.
